# No differences in objective dynamic instability during acceleration of the knee with or without subjective instability post-total knee arthroplasty

**DOI:** 10.1371/journal.pone.0194221

**Published:** 2018-03-16

**Authors:** Tatsuya Soeno, Tomoharu Mochizuki, Osamu Tanifuji, Hiroshi Koga, Takayuki Murayama, Hiroki Hijikata, Yuki Takahashi, Naoto Endo

**Affiliations:** 1 Division of Orthopedic Surgery, Department of Regenerative and Transplant Medicine, Niigata University Graduate School of Medical and Dental Science, Niigata, Japan; 2 Department of Orthopaedic Surgery, Niigata Prefectural Central Hospital, Niigata, Japan; University of Memphis, UNITED STATES

## Abstract

**Introduction:**

Instability after total knee arthroplasty is a critical problem. The purpose of this study was to clarify the stability of implanted knees during walking by comparing differences in dynamic instability during knee acceleration between individuals with or without previously experienced subjective instability, as measured by self-reported questionnaire.

**Materials and methods:**

We examined 92 knees with medial pivot implants. Mean patient age and follow-up duration were 78.4 years and 32.8 months, respectively. An accelerometer was used to investigate the accelerations along three axes; that is, vertical (VT), mediolateral (ML), and anteroposterior (AP) directions in 3-dimensional (3D) space. The analysis in the stance phase and gait cycle was performed by: (1) root mean square (RMS) values of acceleration and (2) frequency domain analysis using fast Fourier transformation (FFT). A self-reported knee instability score was used for the subjective feeling of instability.

**Results:**

A total of 76 knees did not feel unstable (group 0), but 16 knees felt unstable (group 1) in patients during activities of daily living. Regarding the RMS, there were no differences in each direction between the groups. For FFT, the cumulative amplitude in the frequency < 30 Hz also showed no significant differences in all directions between the groups during the stance phase (VT, *p* = 0.335; ML, *p* = 0.219; AP, *p* = 0.523) or gait cycle (VT, *p* = 0.077; ML, *p* = 0.082; AP, *p* = 0.499).

**Discussion:**

Gait analysis based on the acceleration data showed that there were no between-group differences in objective dynamic instability during acceleration of the knee, with or without reports of previously experienced subjective instability, as assessed by the self-reported questionnaire.

## Introduction

Instability following total knee arthroplasty (TKA) is a major adverse effect and a common indication for early revision [1–4]. Anteroposterior (AP) instability in the midrange of knee flexion in implanted knees can interfere with activities of daily life, such as walking and going up and down stairs [4, 5]. In patients with TKA, objective AP instability in the midrange of knee flexion is produced by excessive AP laxity. The degree of AP laxity is associated with the subjective instability that is experienced within 3 months post-TKA, as assessed by self-reported questionnaire [5]. When patients with TKA perceive instability during dynamic motion, the occurrence of objective instability is assumed. It is therefore important to detect objective instability in relation to subjective instability.

A dynamic approach addresses the stability system by evaluating the time-dependent structure of data obtained from sources such as fluoroscopy, motion capture systems, and accelerometers [6]. Fluoroscopy via a three-dimensional (3D) to two-dimensional (2D) registration technique provides high accuracy; however, it has limited application because the range of image is narrow and allows only relatively slow motion [7–13]. A motion capture system can capture any movement and is a sensitive method to assess stability in the knees, as the previous studies have reported stability of the osteoarthritic or implanted knees [14–16]. However, the system is expensive and can be operated only in specific facilities.

An accelerometer is an inexpensive and versatile alternative method for analyzing kinematics in 3D space [6, 17–26], while it may be more lack of accuracy than the fluoroscopy and motion capture system. Accelerometers, attached above and/or below the knee, were recently used to study 3D knee motion in patients with knee osteoarthritis (OA) and TKA [19]. The data obtained from a skin-mounted accelerometer during walking was highly reproducible [20]. Accelerometers possess several benefits. Once fitted with an accelerometer, patients are not restricted to a laboratory and can walk with relatively little restriction with small accelerometers. The various accelerometer designs are sensitive and have diverse dynamic ranges [6, 17–19]. Accelerometer-based gait analysis has potential application in rehabilitation and clinical practice settings, as well as in research [25].

The use of time and frequency domain analysis and examination of the time-dependent structure and coupling of segmental accelerations have provided new insight and better understanding of stability, rhythmicity, and smoothness in normal or pathological walking patterns [21–24, 26]. To our knowledge, frequency domain analysis, via fast Fourier transformation (FFT), has never been used to evaluate acceleration in post-TKA. FFT calculates the component frequencies of a signal and the amount of movement (amplitude) at each frequency. The summation of a number of sine and cosine waves of different frequencies and amplitudes generated from the motion of different physical structures can be considered as a frequency signal resulting from ground reaction forces [25]. If the subjects with TKA produce slight motion instability, FFT can detect it as a frequency signal provided by ground reaction forces.

This study aimed to clarify the stability of implanted knees during walking by comparing differences in dynamic instability during knee acceleration between individuals with or without previously experienced subjective instability, as measured by self-reported questionnaire.

## Materials and methods

The institutional review board of Niigata University approved “Three Dimensional Joint Kinematics and Alignment Study” protocol. All subjects provided their written or verbal informed consent to participate in this study. We examined 92 knees (54 females, 72 knees; 16 males, 20 knees) with medial pivot implants, including 37 knees with the ADVANCE^®^ Medial-Pivot Knee system (MicroPort Orthopedics, Inc., Arlington, TN, USA) and 55 knees with the EVOLUTION^®^ Medial-Pivot Knee system (MicroPort Orthopedics Inc.). The two implants are based on an identical concept with single-radius design in the femoral component for restoration of medial pivot motion and AP stability. A total of 19 knees with cruciate-substituting (CS) and 73 with cruciate-retaining (CR) inserts (10–17 mm) were used. The posterior cruciate ligament was preserved for CR inserts, but not for CS inserts. The mean age, evaluation time, and body mass index were 78.4 years [95% confidence interval (CI), 76.6–80.2], 32.8 months (95% CI, 26.9–38.7), and 25.3 kg/m^2^, (95% CI, 24.5–26.1), respectively.

The inclusion criterion consisted of patients who underwent primary TKA for varus knee OA. Exclusion criteria were valgus knee OA, inflammatory disease, rheumatoid arthritis, secondary OA after trauma, and any indication other than primary arthroplasty, such as high tibial osteotomy or revision arthroplasty.

To simulate the size and setting position of the femoral and tibial components in the preoperative planning, we applied a 3D lower extremity alignment assessment system (Knee CAS; LEXI, Inc., Tokyo, Japan) [27–31] and 3D preoperative planning software (JIGEN; LEXI, Inc.). On the femoral side, we used an intramedullary alignment rod and anterior reference cutting technique. The distal cutting surface was perpendicular to the mechanical axis. The rotational alignment of the femoral component was based on epicondylar axes. The proximal tibia was resected at 90° to the longitudinal axis of the tibial shaft in the coronal plane. The tibial posterior slope was 3° or dependent on the preoperative angle to the longitudinal axis of the tibial shaft in each case.

We used a self-reported knee instability score to measure the subjective instability [5, 32]. Instability was defined as the sensation of buckling or shifting or the knee giving way. Each subject recorded his or her number of instability episodes during the previous 3 months. The questionnaire additionally recorded each subject’s history of falls due to instability and activities associated with instability, such as walking, sitting down or rising from a chair, twisting or turning, ascending or descending stairs, and other activities. Based on their questionnaire responses, subjects were classified into instability or non-instability groups.

An accelerometer (WAA-10; ATR-Promotions, Inc., Kyoto, Japan) was used to investigate the accelerations along three axes, vertical (VT; positive superiorly), mediolateral (ML; positive medially), and anteroposterior (AP; positive anteriorly), in 3D space with a sampling rate of 250 Hz. The rubber strap was firmly attached at the anterior aspect of the tibial tubercle in the proximal tibia, with no intervening clothing. The examiners placed the accelerometer as closely as possible to the bone (the top of the tibial tubercle) to align the accelerometer axes. Khan et al. [19] reported high reproducibility of a skin-mounted accelerometer with a rubber strap during walking. The subjects walked at a comfortable speed for 4–10 meters. The mean (± SD) walking speed was 2.65 ± 0.73 m/s, and there were no differences between the two groups (non-instability group: 2.68 ± 0.74 m/s; instability group: 2.52 ± 0.67 m/s). The accelerometer data were automatically entered into the software (AccelViewer Hyblid; Wireless Technologies, Inc., Tokyo, Japan) on a computer via the wireless system. The points of heel strike and toe-off were determined according to the report by Salles et al. [33], which assessed one gait cycle using the VT direction; this information was applied to the ML and AP directions.

In the root mean square (RMS) values of acceleration, the average of three strides of a stance phase and gait cycle was selected for analysis. To normalize the data, each gait cycle was divided into 100 data points and quantitatively analyzed. RMS was normalized by square of walking speed in each subject. RMS is a measure of dispersion of the data relative to zero, as opposed to the standard deviation, which is a measure of dispersion relative to the mean. This value indicates the average magnitude of accelerations in each direction during walking [23].

FFT is a method of calculating the amount of movement (amplitude) at each frequency. The cumulative amplitude was determined by cumulatively summing the values at each frequency [26]. FFT was applied by the raw accelerometer output prior to analysis. In this (FFT) analysis, we used 512 sampling points of two multipliers from the raw acceleration data, in all directions (VT, ML, and AP). When the raw acceleration data number was not a two multiplier point, the missing point was revised with 0. FFT converted the acceleration raw data into complex values. The cumulative amplitude of the different frequencies < 30 Hz was obtained through FFT analysis.

These data were compared between the instability and non-instability groups. Further, we compared each parameter among four groups with or without subjective instability in the contralateral or bilateral implanted knees.

### Statistical analysis

The distribution and variance were examined using a Shapiro–Wilk test and Levene test, respectively. We statistically compared each demographic data parameter (Table 1) and RMS (Table 2) between instability and non-instability groups using a two sample t-test when the data had a normal distribution and equal variances. A Mann–Whitney test was used when the data did not have a normal distribution. A chi-square test was used to compare group proportions in the demographic data. The comparison of RMS among the four groups divided by the contralateral and bilateral implanted knees, with or without subjective instability, was evaluated by one-way analysis of variance (ANOVA) when the data had a normal distribution and by Kruskal–Wallis test when the data did not have a normal distribution (Table 3). In the analysis of FFT data, repeated-measures ANOVA was applied to analyze qualitative variables. The effect sizes are shown in Tables 2 and 3. These parameters were analyzed using SPSS, version 21 (SPSS, Inc., Chicago, IL, USA). The statistical significance was set at *p* < 0.05. All data in this study were shown in S1 Table.

**Table 1:** The demographic data.

Variable	Subjective instability (-) n = 76		Subjective instability (+) n = 16		
	mean	95%CI	mean	95%CI	*p* value
Age (years)	79.3	77.5–81.0	74.2	68.2–80.1	0.166
BMI (kg/m^2^)	25.6	24.7–26.5	23.9	22.8–25.0	0.137
Walking speed(m/s^2^)	2.68	2.51–2.85	2.52	2.19–2.85	0.429
Evaluation time post-TKA (months)	33.8	27.0–40.4	28.3	16.5–40.0	0.348
Gender					
Female (n)	58		14		0.306
Male (n)	18		2		
Implant—insert					
EVOLUTION–CR (n)	34		9		0.164
EVOLUTION–CS (n)	12		0		
ADVANCE–CR (n)	23		7		
ADVANCE–CS (n)	7		0		

CR = cruciate retaining insert; CS = cruciate substituting insert; 95%CI = 95% confidence interval; n = knees.

**Table 2:** The root mean square (RMS) between the groups with and without subjective instability.

	Subjective instability (-)		Subjective instability (+)			
	mean	95%CI	mean	95%CI	effect size (r)	*p* value
VT (S)	188.6	152.4–224.9	172.3	121.9-222-7	0.04	0.578
VT (G)	173.7	139.5–208.0	158.6	111.0–206.2	0.04	0.543
ML (S)	43.8	33.6–54.0	36.8	25.6–48.0	0.06	0.757
ML (G)	47.9	36.9–58.8	41.7	25.3–58.1	0.05	0.543
AP (S)	61.0	48.4–73.7	54.2	39.5–68.8	0.05	0.703
AP (G)	85.6	69.9–101.4	77.2	59.8–94.6	0.05	0.578

95%CI = 95% confidence interval; VT = vertical; ML = mediolateral; AP = anterior-posterior; (S) = stance phase; (G) = gait cycle.

**Table 3:** The root mean square (RMS) among four groups.

	Contralateral TKA				Bilateral TKA					
	Subjective instability (-)		Subjective instability (+)		Subjective instability (-)		Subjective instability (+)			
	mean	95%CI	mean	95%CI	mean	95%CI	mean	95%CI	effect size (η^2^)	*p* value
VT (S)	191.4	136.9–245.9	194.5	104.9–284.1	186.0	135.4–236.5	143.7	97.2–190.3	0.01	0.922
VT (G)	176.7	125.5–228.0	178.2	93.0–263.4	170.9	122.8–219.0	133.4	89.4–177.4	0.01	0.919
ML (S)	44.1	28.2–60.0	42.7	23.6–61.8	43.5	29.9–57.1	29.3	17.3–41.3	0.01	0.635
ML (G)	48.0	30.6–65.3	48.7	19.3–78.1	47.8	33.4–62.2	32.7	18.3–47.1	0.01	0.641
AP (S)	60.8	42.9–78.7	61.9	36.2–87.7	61.3	43.0–79.9	44.2	31.7–56.7	0.01	0.827
AP (G)	85.0	62.2–107.8	89.1	61.0–117.1	86.2	63.4–109.1	62.0	43.0–81.0	0.01	0.589

95%CI = 95% confidence interval; VT = vertical; ML = mediolateral; AP = anterior-posterior; (S) = stance phase; (G) = gait cycle.

## Results

**Fig 1:**
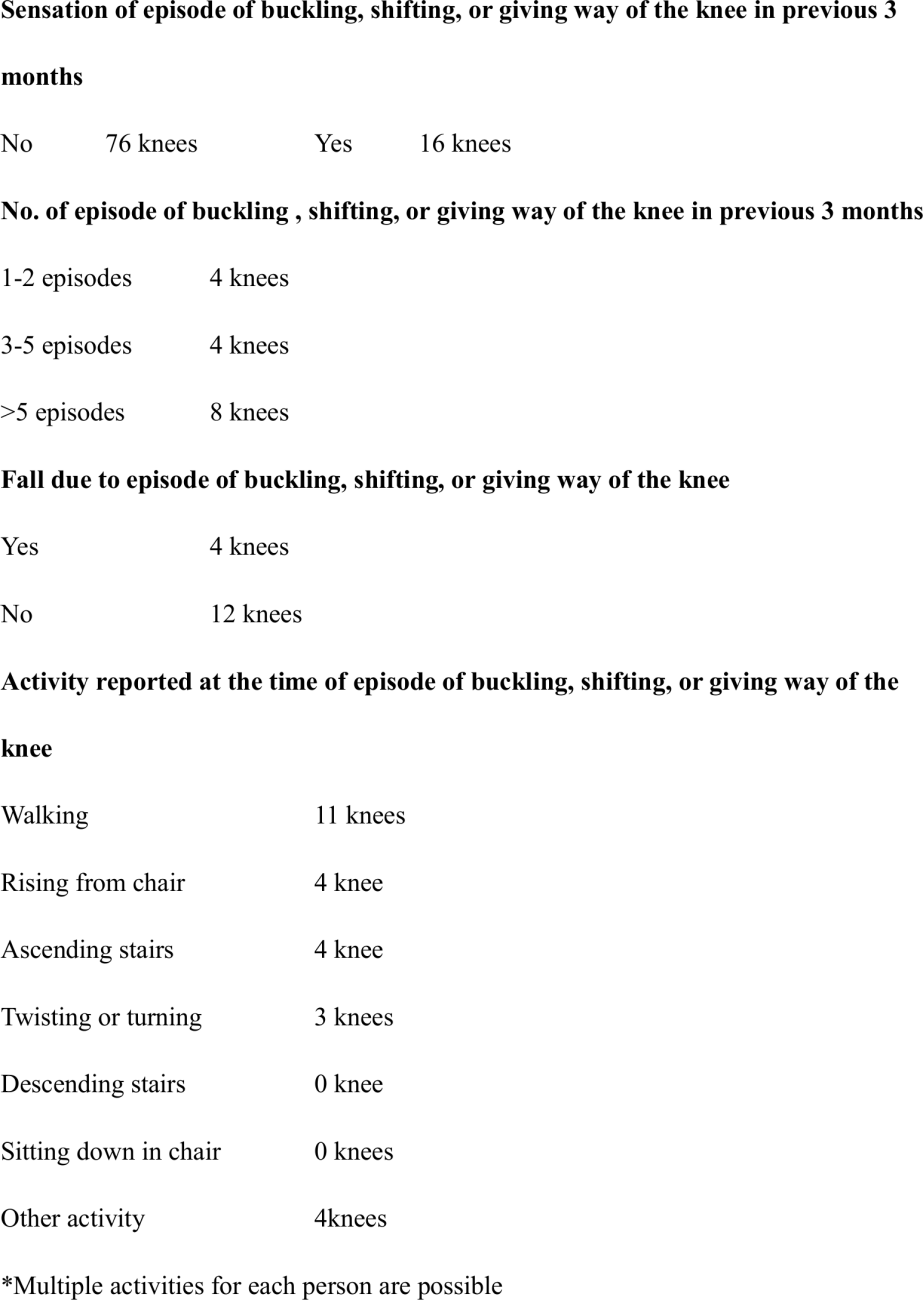
The summary of self-reported knee instability score.

A total of 76 knees did not feel unstable, but 16 knees felt unstable in patients during activities of daily living (Fig 1). Demographic data are shown in Table 1. There were no between-group differences in any parameter (age, BMI, walking speed, evaluation time post-TKA, sex, implant and insert).

**Fig 2:**
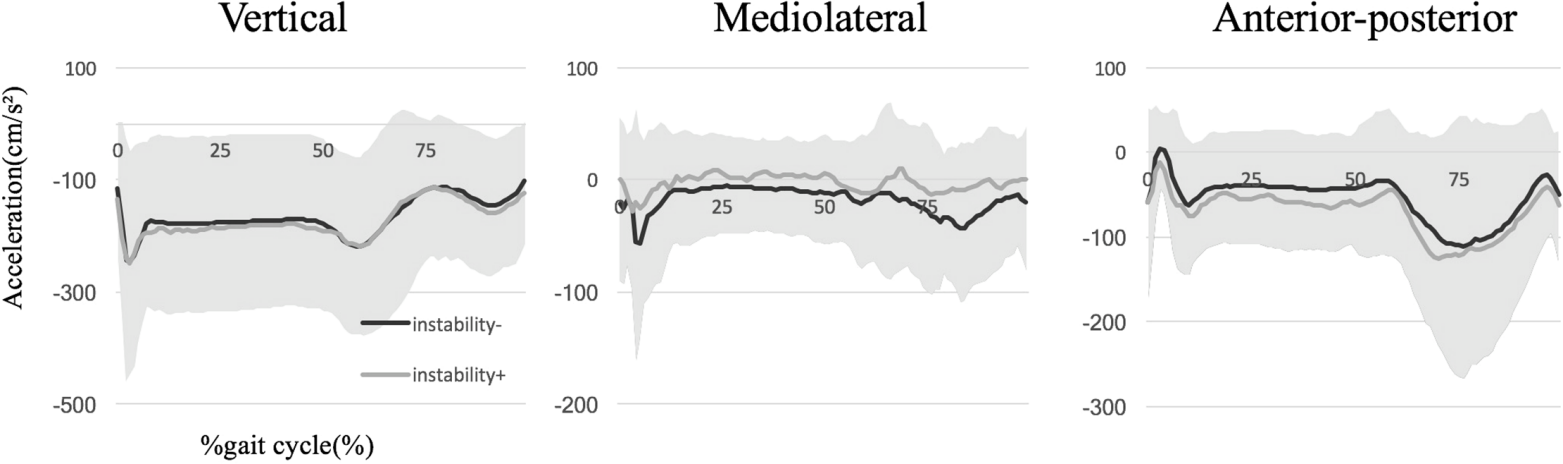
The average wave patterns of accelerations along three axes, including vertical (VT, positive superiorly), mediolateral (ML, positive medially), and anteroposterior (AP, positive anteriorly) directions between the groups with or without subjective instability, are shown. The colored range means standard deviation (SD) in all groups.

**Fig 3:**
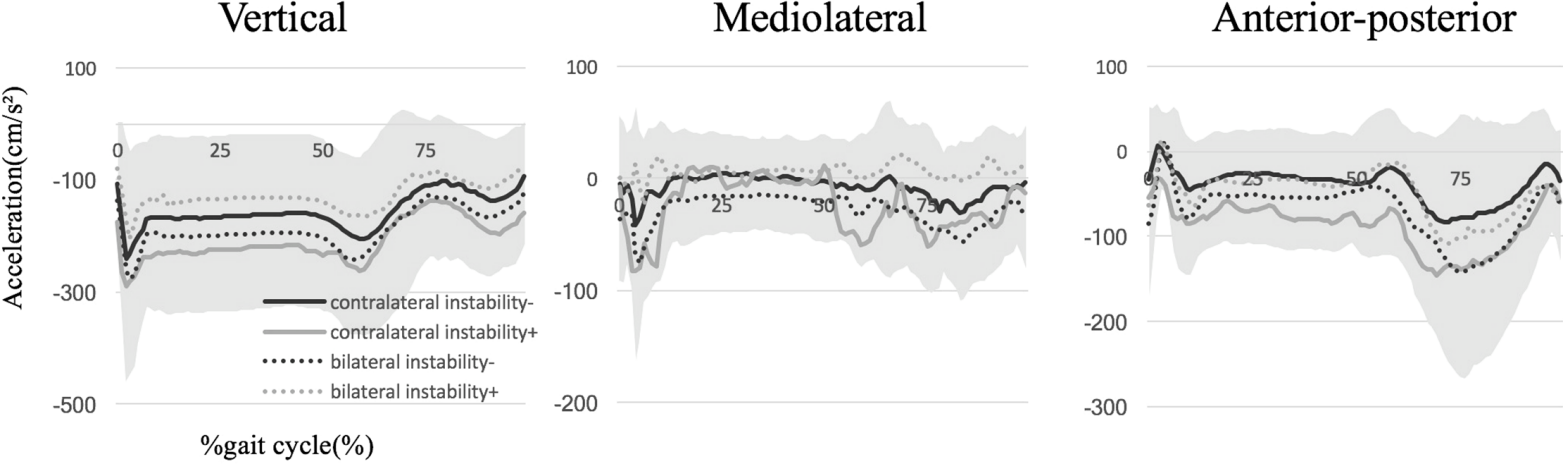
The average wave patterns of accelerations along three axes, including vertical (VT, positive superiorly), mediolateral (ML, positive medially), and anteroposterior (AP, positive anteriorly) directions among four groups with or without subjective instability in the contralateral or bilateral implanted knees, are shown. The colored range means standard deviation (SD) in all groups.

Regarding RMS in the average of three strides, there were no between-group differences in any direction, with or without subjective instability during the stance phase (VT, *p* = 0.578; ML, *p* = 0.757; AP, *p* = 0.703) or gait cycle (VT, *p* = 0.543; ML, *p* = 0.543; AP, *p* = 0.578) (Table 2) (Fig 2). Comparing four groups including those who received contralateral or bilateral TKA, with or without subjective instability, there were no between-group differences in any direction during the stance phase (VT, *p* = 0.922; ML, *p* = 0.635; AP, *p* = 0.827) or gait cycle (VT, *p* = 0.919; ML, *p* = 0.641; AP, *p* = 0.589) (Table 3) (Fig 3).

**Fig 4:**
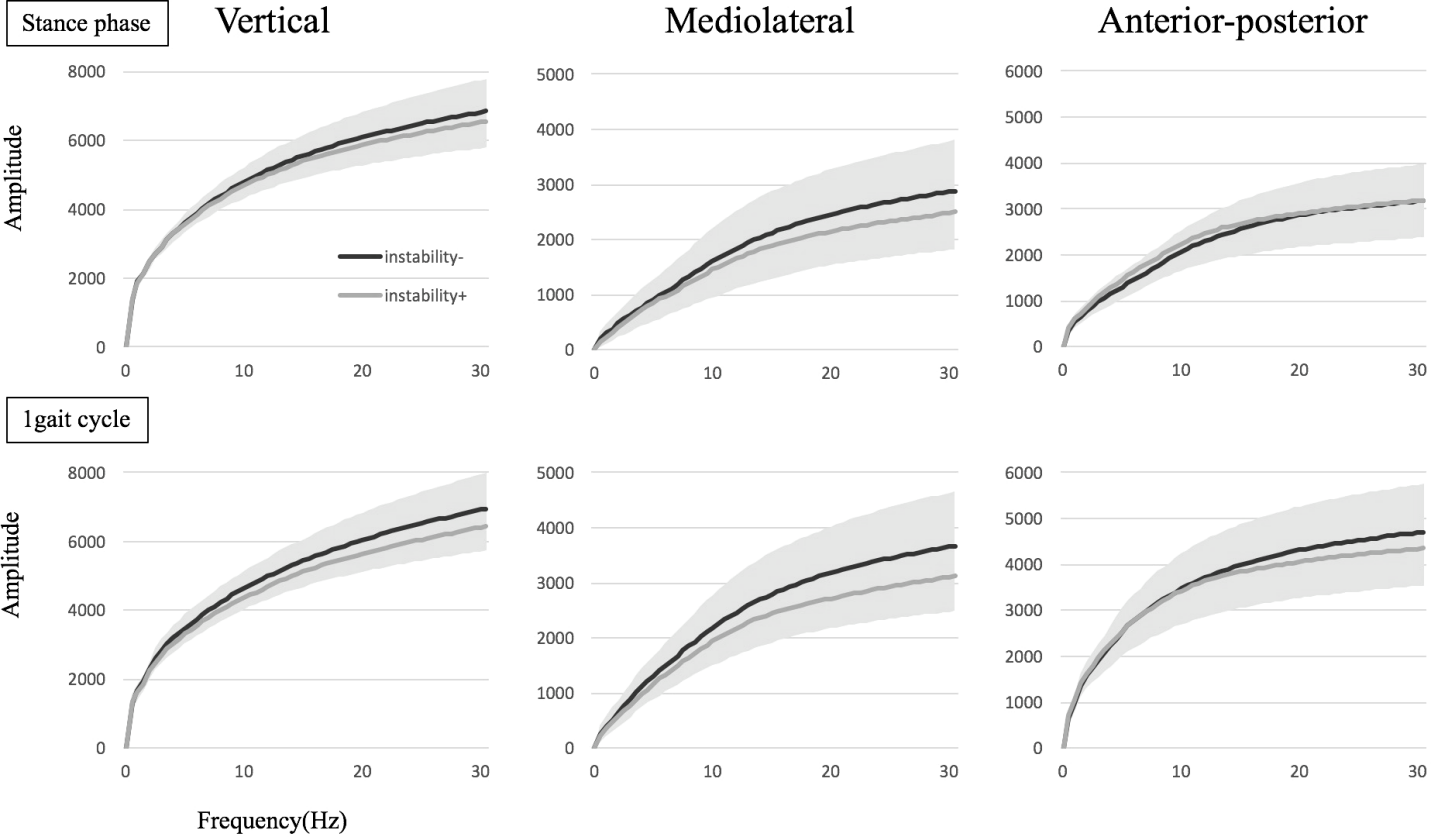
The cumulative amplitude in the frequency < 30 Hz in the fast Fourier transform (FFT) between the groups with or without subjective instability is shown. The colored range means standard deviation (SD) in all groups.

**Fig 5:**
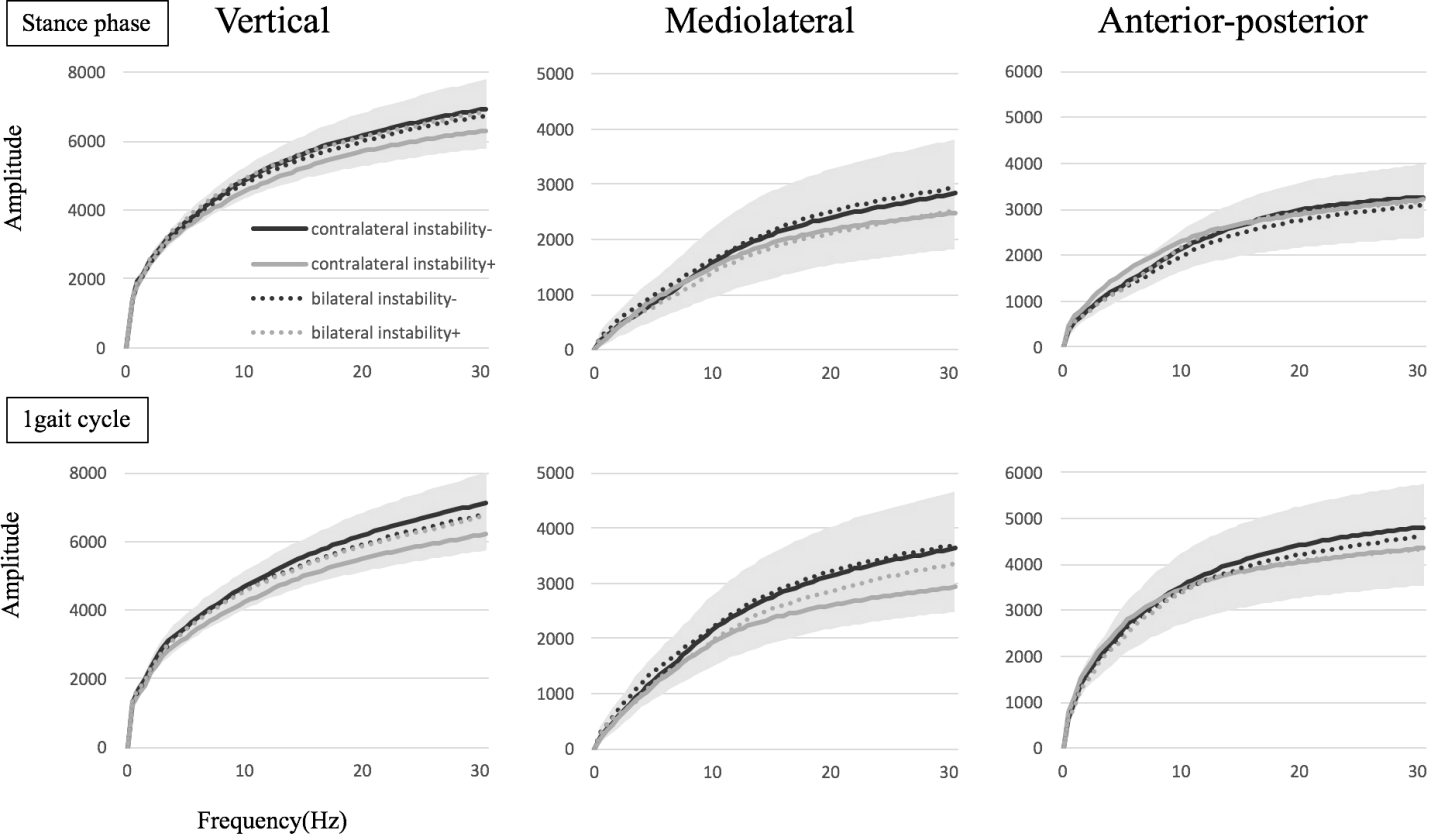
The cumulative amplitude in the frequency < 30 Hz in the fast Fourier transform (FFT) among four groups with or without subjective instability in the contralateral or bilateral implanted knees is shown. The colored range means standard deviation (SD) in all groups.

For FFT, the cumulative amplitude in the frequencies < 30 Hz also showed no significant between-group differences in any direction, with or without subjective instability during the stance phase (VT, *p* = 0.335; ML, *p* = 0.219; AP, *p* = 0.523) or gait cycle (VT, *p* = 0.077; ML, *p* = 0.082; AP, *p* = 0.499) (Fig 4) and among four groups divided by the contralateral or bilateral TKA with or without subjective instability during the stance phase (VT, *p* = 0.311; ML, *p* = 0.590; AP, *p* = 0.535) or gait cycle (VT, *p* = 0.123; ML, *p* = 0.315; AP, *p* = 0.768) (Fig 5).

## Discussion

There were no differences in dynamic instability, as indicated by acceleration of the knee, between the groups with or without subjective instability, as measured by self-reported questionnaire.

Frequency domain analysis of the acceleration signal adds to our understanding of how the locomotor system organizes movement [6, 26]. FFT calculates the component frequencies of a signal and the amount of movement (amplitude) at each frequency. The summation of a number of waves of different frequencies and amplitudes created from the movement of different anatomical structures can be assumed as a frequency signal resulting from ground reaction forces [25]. Staab et al. [25] showed the difference in spectral visualization of normal human gait derived from measurements by a 3D accelerometer via FFT compared with an OA knee. Kavanagh et al. [26] investigated the age-related differences in the head and trunk using frequency domain analysis, and showed that compared with the young, the elderly had a greater proportion of power in the trunk (>6 Hz). They subsequently concluded that the elderly, compared with the young, are confronted with different and more challenging postural conditions during which they must maintain dynamic stability.

TKA instability in the AP direction due to excessive laxity during extension to midrange flexion is a critical problem. This laxity can cause instability and pain, thereby leading to revision surgeries [4]. The presence of a posterior cruciate ligament (PCL) post-TKA, with CR procedures, cannot provide AP stability from extension to midrange flexion. PCL is not tight until at least 45° of flexion; ligaments that are lightly tensed cannot prevent forward sliding [34]. Thus, the shape of the component and inserts are important factors for preventing AP instability throughout the range of motion [34, 35]. The medial pivot design theoretically mimics the normal knee kinematics of medial pivot motion to achieve AP stability [34, 35]. This study demonstrated that there were no between-group differences in dynamic instability in any direction, including the AP direction, during acceleration, with or without subjective instability. The implanted knees might have prevented objective dynamic instability by acceleration in the WB condition because of the medial pivot design; however, this is just a speculation. The discrepancy between dynamic instability during acceleration and previously experienced subjective instability is a necessary topic of discussion. The possible reasons for this discrepancy include the following: (1) subjective instability might occur without dynamic instability in the implanted knees, (2) subjective instability might not occur because dynamic instability did not appear at the time of the measurement, regardless of the potential for dynamic instability in the implanted knees. Regarding reason (1), the compensatory mechanisms of the human body could be insufficient. Regarding reason (2), the compensatory mechanism might be sufficient. Dubousset [36] described the “cone of economy” using the concept of optimal posture and standing balance. In the cone, the body can remain balanced without external supports using minimal effort. Skoyles [37] noted that, when the body’s center of mass relocates over the foot support, human vertical balance will synchronize any action with a skeletomuscular adjustment that corrects for its potential destabilizing effects. Shibasaki et al. [38] reported that the final common pathway for gait is formed by neuromuscular function and feedback receptive systems, such as the somatosensory, vestibular, visual, and auditory systems. Many studies have showed associations between changes in gait and the risk of falling in elderly subjects [39, 40]. Gait changes that prevent falling likely increase stability during walking [41]. In view of the potential influence of subjective instability on motor behaviors, it is likely that the neuromuscular control and feedback systems compensate for dynamic instability to achieve stability.

In this study, subjects with excessive AP laxity in non-WB conditions post-TKA had a more stable knee in the AP direction during gait-related WB conditions. This phenomenon may occur because of the function of extensor and flexor muscles around the knee, other neuromuscular supports, and the ability for human vertical balance processes to compensate for excessive AP laxity, possibly causing subjective instability. The subjects in the present study perceived sudden instability when they were not careful with their daily movements. Under gravity, the neuromuscular control and feedback systems for achieving and maintaining stability are important during daily activities; hence, when these functions are inefficient, patients with TKA may suddenly perceive instability, even if the implanted knees possess adequate tension.

There are several limitations to the current study. First, the two implants were similar, each having a single-radius design for restoring medial pivot motion and AP stability. Other implants with different designs may produce different results; this is the future direction of our research. Second, this study measured only simple gait. We did not measure movements associated with going up and down stairs or other activities of daily life. Other actions aside from gait might produce objective dynamic instability by acceleration. However, since the acceleration provided by the impaction at heel strike might be too strong during stair climbing and other such activities, the accelerometer data might be unreliable in these situations. Third, accelerometers move on the skin, not on the bone, and this discrepancy might substantially affect the accuracy of the data they record. However, Khan et al. [19] showed high reproducibility of an accelerometer that was skin-mounted with a rubber strap during walking; therefore the reliability of the data generated during this study can be reasonably assumed. Fourth, ideally, the RMS should be normalized after calculating the accurate relationship between the acceleration and velocity because of non-linear relationship, but it was impossible in this system. As the previous study reported that the relationship between the acceleration and velocity was exponential [23], the data was normalized by square of the velocity in the present study.

## Conclusions

Gait analysis based on the acceleration data showed that there were no between-group differences in objective dynamic instability during acceleration of the knee, with or without reports of previously experienced subjective instability, as assessed by the self-reported questionnaire.

## Supporting information

S1 Table All data in this study are included. (XLSX)
